# Phenotypic plasticity or speciation? A case from a clonal marine organism

**DOI:** 10.1186/1471-2148-8-47

**Published:** 2008-02-13

**Authors:** Carlos Prada, Nikolaos V Schizas, Paul M Yoshioka

**Affiliations:** 1 Department of Marine Sciences, University of Puerto Rico-Mayagüez, Isla Magueyes Laboratories, P.O. Box 908, Lajas, PR 00667, USA

## Abstract

**Background:**

Clonal marine organisms exhibit high levels of morphological variation. Morphological differences may be a response to environmental factors but also they can be attributed to accumulated genetic differences due to disruption of gene flow among populations. In this study, we examined the extensive morphological variation (of 14 characters) in natural populations observed in the gorgonian *Eunicea flexuosa*, a widely distributed Caribbean octocoral. Eco-phenotypic and genetic effects were evaluated by reciprocal transplants of colonies inhabiting opposite ends of the depth gradient and analysis of population genetics of mitochondrial and nuclear genes, respectively.

**Results:**

Significant differences (P < 0.001) in 14 morphological traits were found among colonies inhabiting 12 locations distributed in seven reefs in southwest Puerto Rico. Results from principal component analysis indicated the presence of two groups based on depth distribution, suggesting the presence of two discrete morphotypes (i.e. shallow type < 5 m and deep type > 17 m). A discriminant function analysis based on a priori univariate and multivariate analyses (which separated the colonies in morphotypes) correctly classified 93% of the colonies for each environment. Light, water motion and sediment transport might influence the distribution of the two morphotypes. Reaction norms of morphological characters of colonies reciprocally transplanted showed gradual significant changes through the 15 months of transplantation. Sclerites of shallow water colonies became larger when transplanted to deeper environments and vice versa, but neither of the two transplanted groups overlapped with the residents' morphology. Genetic analysis of mitochondrial and nuclear genes suggested that such discrete morphology and non-overlapping phenotypic plasticity is correlated with the presence of two independent evolutionary lineages. The distribution of the lineages is non-random and may be related to adaptational responses of each lineage to the environmental demands of each habitat.

**Conclusion:**

The extensive distribution and ample morphological variation of *Eunicea flexuosa *corresponds to two distinct genetic lineages with narrower distributions and more rigid phenotypic plasticity than the original description. The accepted description *sensu *Bayer (1961) of *E. flexuosa *is a complex of at least two distinct genetic lineages, adapted to different habitats and do not exchange genetic material despite living in sympatry. The present study highlights the importance of correctly defining species, because the unknowingly use of species complexes can overestimate geographical distribution, population abundance, and physiological tolerance.

## Background

The phenotype is considered the product of inherited genetic information and its interaction with the environment. Thus, differences in the phenotype can be explained by variations in environmental conditions, but also such phenotypic differences may reflect accumulated genetic variation due to disruption of gene flow between populations, and their subsequent speciation into biological species.

First, phenotypic plasticity enhances the survival and reproductive success of individuals by contributing to their ability to cope with environmental changes and to potentially adapt to new niches. Plasticity is an emergent property of the genotype and therefore also susceptible to natural selection [1]. The change of the plastic response is often continuous, when the trait under analysis is subjected to an environmental gradient suspected to induce changes [2]. The spectrum of phenotypes due to the environmental change describes the norms of reaction [2,3]. Among the metazoans that exhibit the most extensive phenotypic plasticity are the marine modular species.

Phenotypic plasticity has been studied in algae [4], sponges [5], barnacles [6], gastropods [7,8], bryozoans [9] and anthozoans [10-16]. This plasticity provides organisms with the ability to generate the fittest phenotype suiting local conditions. Morphology is then acquired through development under the current environment and can be changed in the next generation, if conditions are modified. Strong environmental gradients in the sea (e.g. light, water flow, sediment transport) may restrict the distribution of individuals to habitats, representing opposite ends of the gradient, where each phenotype is adapted [17,18]. Furthermore, the fitness of the phenotypes varies along the environmental gradient [17]. Disruptive selection may enhance the success of the two phenotypes at the opposite ends of the gradient by ecologically favoring each phenotype in its more suitable environment and by increasing genetic divergence. In this case, organisms settle and suffer high mortalities in non-optimal environments. Disruptive selection may be an influential evolutionary force leading to two disparate phenotypes by the existence of non-random mating related to habitat utilization [19].

In the absence of local adaptation, the high dispersal potential of marine propagules usually results in genetic homogeneity over large distances [20-22]. However, allopatric speciation is possible mainly because changes in oceanographic conditions, the emergence of land masses [23], and disconnection of populations by lower sea levels [24]. As gene flow is disrupted by a geographic barrier, populations become isolated and diverge due to genetic drift. After genetic divergence has been acquired through generations of genetic drift and restricted gene flow, secondary contact can be achieved when the two new lineages attain similar geographic distributions [25].

Apart from allopatric divergence, sympatric divergence is also plausible. Speciation has occurred in spawning organisms with larvae capable of long dispersal [26-28] and genetic differences have been detected in sympatric populations [29,30]. Ecological specializations to different habitats [31,32], variable symbiotic relationships related to habitat distribution [33] and unsynchronized gamete release [34-36] may prevent organisms to reproduce randomly in sympatry, leading to a rapid evolution of mating systems [37-39] and eventually to speciation. It is not surprising that sibling species in the sea are more common than previously thought [40]. Species with novel gene combinations can also be formed sympatrically through hybridization, an important process of diversification in marine and terrestrial systems [41-43].

In octocorals, phenotypic plasticity along environmental gradients or habitats is not uncommon [12,14,29,44-47]. Octocorals are relatively abundant and visually dominant in low relief hard ground habitats with preference for high water motion areas [48,49]. Light, water motion and sediment transport are determining factors in the distribution of gorgonians [49]. These abiotic factors may induce morphological adjustments in broadly distributed species to optimize fitness under suboptimal conditions. Colonies of *Eunicea flexuosa *(Lamouroux 1821), in shallow forereef areas are susceptible to high water motion and are generally taller; grow in a single plane with thicker branches and bigger calices. In contrast, colonies in deeper environments are exposed to low water motion and less light. There, the colonies exhibit multiplane growth, are smaller with fewer terminal branches than their shallow counterparts with smaller and more sparse calices [12]. Sclerite plasticity has also been correlated with differences in water motion and light [12,14]. Smaller and thinner clubs and spindles are present in high water motion environments (i.e., forereef areas), providing a stronger structure and support to the colony [14]. The high morphological variability in *E. flexuosa *could be due to phenotypic plasticity, genetic differentiation or a combined effect. In this study, first, the morphological variation of 14 traits of *E. flexuosa *was evaluated in seven reefs (from protected to exposed areas to water motion) at two depths (< 5 m and >17 m) and the correlation of morphology with light, water motion and sediment patterns was inferred. Second, environmental and/or genetic factors were studied to define the morphological variation of *E. flexuosa*. Reciprocal transplants of colonies inhabiting the opposite ends of the depth gradient were used to infer patterns, magnitude and direction of the phenotypic response. Gene genealogies of the mitochondrial gene *msh1 *and the nuclear gene 18S were used to elucidate possible genetic-phenotypic interactions. Lastly, allopatric and sympatric divergence (through ecological differentiation) and hybridization was considered as possible evolutionary processes that produce and maintain the morphological and genetic variation found in *E. flexuosa*.

## Results

### Natural Variability of Morphological Traits

**Figure 1:**
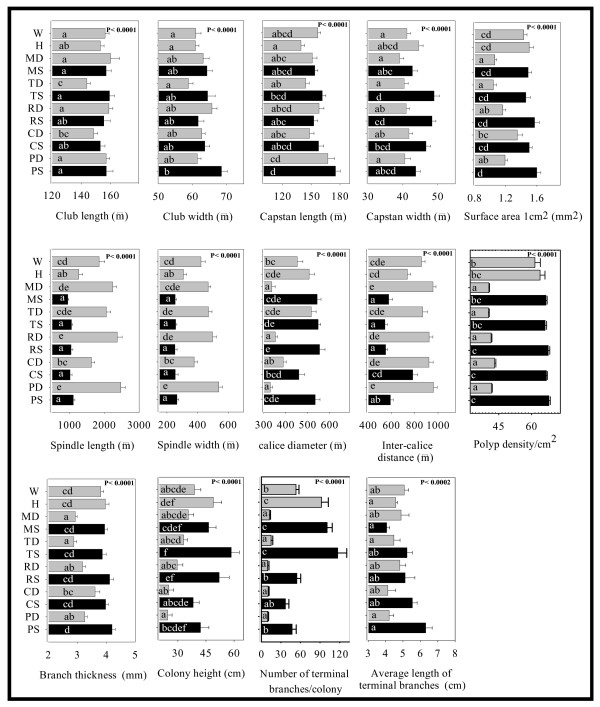
Extent of variation in phenotypic traits analyzed by individual nested ANOVAs for 14 morphological characters. Colonies are nested within habitats. Black and white bars represent samples in shallow and deep areas, respectively. Values are shown as mean value ± SE (n = 300 measurements per location for micromorphological characters and 15 per location for macromorphological characters). Lowercase letters indicate treatments that are statistically different by post hoc analysis. The location codes are as in Table 6.

Mean values per trait and habitat are shown in Fig. 1. Measurements of morphological characters of most colonies in a given location fell within a narrow range around the mean. However, there were few colonies that did not match the mean population values. These atypical colonies were closer morphologically to colonies inhabiting the opposite depth habitat than to their neighbours and showed that in a given location, colonies under similar environmental conditions develop different morphologies. All traits varied significantly between reefs and among depths (Fig. 1). Values for CL, CW, CAL, CAW CD, SA, BT, CH, PD, TBN, and TBM decreased as depth increased, whereas values for SL, SW and ID increased with increasing depth (Fig. 1).

Depth differences were also recovered in the principal component analysis. The first principal component (PC1) explained 64% of the variance among measurements and was characterized by high negative loadings for SL, SW and ID and high positive loadings for CW, CAW, SA and BT (Table 1). PC2 explained 16% of the variance and was characterized mainly by positively weighted CL, CW and CAL and negatively by CD. Two-way ANOVA on the loading values of the first three principal components showed significant differences in reef and depth in PC1 (Table 2) and PC2 loadings showed significant differences in reef. Since 80% of the variation was explained by the first two principal components, environmental gradients related to depth, water motion, light and sediment transport could probably explain a portion of the morphological differences. As these co-vary with depth, it was not possible to quantify their individual effects on the morphological variation of *E. flexuosa*.

**Table 1:** Summary results of the principal component analysis.

Characters	PC1	PC2	PC3
**Eigenvalue**	7.67	1.87	0.93
**Explained Variance (%)**	64	16	8
**CL**	0.14	0.43	0.60
**CW**	0.26	0.36	-0.07
**CAL**	0.17	0.53	0.01
**CAW**	0.27	-0.10	-0.01
**SL**	-0.31	0.18	0.19
**SW**	-0.31	0.13	0.16
**CD**	0.24	-0.37	-0.04
**ID**	-0.31	0.13	-0.15
**SA**	0.30	0.09	-0.27
**BT**	0.30	0.09	-0.27
**TBM**	0.20	0.32	-0.21
**TBN**	0.27	-0.23	0.42
**CH**	0.27	-0.17	0.43
**PD**	0.32	-0.01	-0.05

The first three principal components are shown, calculated from 14 skeletal traits measured on *E. flexuosa*, collected from the 12 locations (seven reefs were included). The character codes are: club length (CL), club width (CW), capstan length (CAL), capstan width (CAW), spindle length (SL), spindle width (SW), calice diameter (CD), intercalice distance (ID), surface area (SA), polyp density (PD), branch thickness (BT), terminal branch number (TBN) terminal branch average length (TBL) and colony height (CH).

**Table 2:** Two-way ANOVA results showing the effects of depth, zone and the interaction between depth and zone on the first three principal components.

Parameter	df	PC1		PC2		PC3	
		
		MS	F	MS	F	MS	F
**Zone**	1	0.044	0.279	3.238	3.415	11.553	13.571***
**Reef(Zone)**	2	0.826	5.198***	3.755	3.96*	0.328	0.385
**Depth**	1	99.079	623.196***	0.087	0.092	0.029	0.034
Zone*Depth	1	0.100	0.628	0.074	0.078	9.721	11.42***
**Error**	114	0.159		0.948		0.851	

The PCA loading scores were obtained from analysis of 12 morphological features of *E. flexuosa *in the sampling environments. Note that only eight of the 12 locations were included to achieve a balanced design. Significant values are represented by ***, ** and * for *P *< 0.001, *P *< 0.01 and *P *< 0.05, respectively.

**Figure 2:**
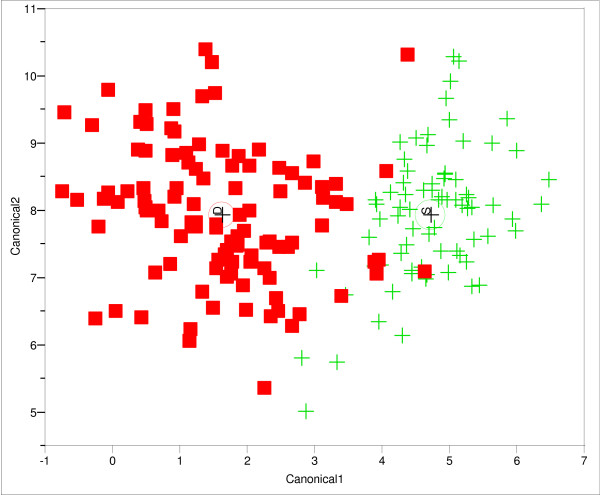
Discriminant function analysis plot based on 14 morphological characters of *E. flexuosa *colonies inhabiting different depth habitats (D for deep and S for shallow). Multivariate comparison (fixed-effect MANOVA) among depths was significant (Wilks' λ = 0.2769; F = 30.7713 df = 14/167; *P *< 0.001). Misclassified colonies = 14 (7%). The canonical axis 1 mostly weighted by length and width of spindle and club, intercalice distance surface area and branch thickness, while canonical axis 2 was mostly weighted by width and length of clubs and calice density.

Results of the discriminant function analysis based on Wilk's λ (depth = 0.19, F = 56.29, *P *< 0.0001; reef λ = 0.31, F = 3.60, *P *< 0.0001 and zone λ = 0.21, F = 15.76, *P *< 0.0001) showed significant differences in colonies of different depths, reefs and zones. Depth differences as expected from the univariate and PCA analyses were clearly defined (Fig. 2) and the correct classification percentage was high (93%). The colonies in shallow habitats are concentrated on the right side of the canonical plot, while colonies in deep areas are mostly in the left side. There were a few deep water colonies misclassified and embedded within the shallow ones and vice versa (Fig. 2). Canonical plots of the DFAs of reef and zone showed high percentages of misclassification, 39% and 41%, however, a tendency from protected and deep to exposed and shallow was observed (data not shown).

**Figure 3:**
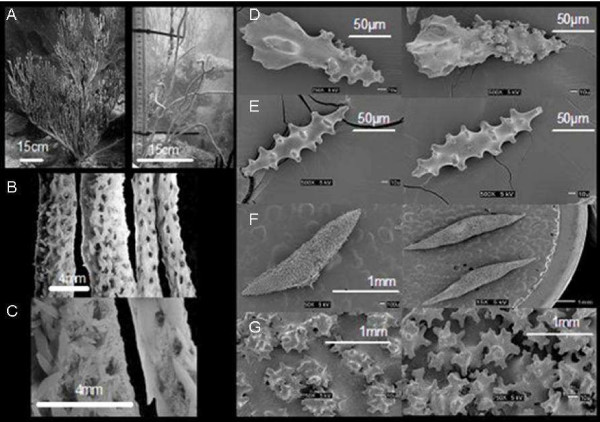
Differences among colonies inhabiting deep and shallow areas. A. Colonies in shallow areas (left), compared to deep colonies (right) are more branched and bigger in size. Thicker colonies are found in shallow areas with smaller spindles and more polyps per area (B and C). Right showing differences among sclerites; club (D), capstan (E), spindle (F) and spindle close up (G) of colonies inhabiting shallow (left) and deep (right) areas. Spindles of shallow area colonies compared to deep colonies are bigger in size. The surface arrangements of warts are sparser in shallow area colonies (G).

The results suggest the presence of two morphotypes (Fig. 3). The first morphotype fits well with Bayer's description; adult colonies exhibit bushy-like shape and branch profusely in a single plane. Microscopically, size of leaf clubs, structural spindles and fused capstans are of ~200 μm, ~1000 μm and ~200 μm, respectively. The two morphotypes found in two depth habitats (hereafter deep and shallow indicating the depth of habitat) are exposed to different water motion, light, and sediment transport regimes. However, each morphotype was recorded in very low numbers at the opposite habitat. The shallow type was found more frequently in deep habitats than the deep type in shallow areas. Also, colonies sampled in the outer shelf reefs were morphologically closer to those in shallow areas of inshore and mid reefs; however the presence of both morphotypes was higher in such environments and each type was represented by at least six colonies (15 colonies total). In Weinberg, the presence of deep type colonies was associated with sand channels, areas characterized by high sediment transport. Along these channels deep type colonies were more frequently encountered.

### Transplant Experiment

On average, transplanted branches grew 1.94 ± 0.34 (1 SD) cm during the 15 months of the study. The new tissue deposited at the tip of the branches was enough to allow sampling of sclerites developed under novel conditions, thus the analysis excluded premature sclerites present at the very tip of the colony.

A two-way ANOVA test on linear growth values revealed a significant difference across depths (df = 1, F = 12.15, *P *= 0.001). Colonies in shallow environments regardless of population source (residents or transplanted) grew almost twice as fast as their deep counterparts. Population source and population X depth interaction were not significant (df = 1, 1; F = 0.067, 0.10; *P *= 0.80, 0.75; respectively). Of the 90 initial colonies transplanted, 59 were recovered for sclerite analysis. The mortality was not independent among groups (X^2 ^= 10.449, df = 3, *P *< 0.025). The control colonies either from deep to deep, or shallow to shallow had higher survivorship (93% and 80%, respectively) than the transplanted ones. Mortality was highest (57% ex 17 of 30 colonies) in colonies transplanted from shallow to deep areas. Most of these colonies died by the 6^th ^month; however neither detachment nor presence of predators was noticeable during the experiment. Competition with other reef organisms was also not evident. In most cases the tissue started to peel away until the entire axis was exposed. Colonies transplanted from deep to shallow areas had high survivorship (77% ex 23 of 30 colonies), nonetheless bleaching was recorded in 14 colonies (46%) and 23% of those died.

**Figure 4:**
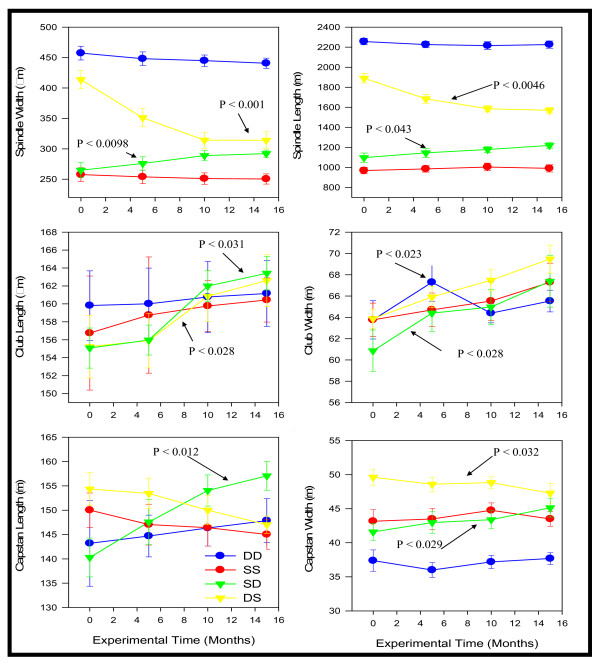
Reaction norms of morphological traits through time in transplanted colonies. Spindle changes are more drastic than club and capstans. Population codes are SS: Shallow to shallow; DD: Deep to deep for residents and SD: Shallow to deep and DS: Deep to shallow for reciprocal transplants. Values are given as mean value ± 1 standard deviation.

After 15 months of the experiment, measurements of sclerites for shallow and deep colonies were within the range suggested by the natural variability analyses (Fig. 4). However, spindle width and length of the reciprocally transplanted colonies gradually became similar to those of the resident colonies but never overlapped, colonies transplanted from shallow to deep areas tended to increase in sclerite size while spindles of colonies from deep to shallow became smaller. CL, CW, CAL and CAW showed similar trends but exhibited lower percent variation within groups, therefore resulting in non-significance between groups comparisons (i.e., DD, SS, SD and DS) (Fig. 4). Some colonies (i.e., SD16, SD18, SD19, DS7, DS8, DS14, DS 15, DS17, DS22, DS 23 and DD2) differed significantly from their source population either shallow or deep. Such atypical colonies did not show the gradual change in spicule size over time, observed within their transplanted group. Two-way repetitive measurement ANOVAs (Table 3) also showed significant differences among populations (DD, SS, SD and DS) for most traits (except CL and CAL) and the time X population interaction in SL and SW.

**Table 3:** Repetitive measurements ANOVAs evaluating the effects of population (transplanted and residents), time (every 5 months until 15 months) and their interaction.

Source of variation	df	SL		SW		CL		CW		CAL		CAW	
	
		MS	F	MS	F	MS	F	MS	F	MS	F	MS	F
**Population**	3	15688573	47.7***	15688573	47.7***	78.207	0.2	271.916	2.8*	197.896	0.5	1140.334	14.6***
**Time**	3	59699.93	1.8	59699.93	1.8	598.211	6.2***	115.605	5.3**	519.5	3.2*	23.082	1.7
**Pop × Time**	9	306976.5	9.7***	306976.5	9.7***	74.681	0.8	37.413	1.7	447.227	2.7**	23.718	1.8
**Total**	259	294507.5		294507.5		144.101		43.889		233.867		42.047	

Significant values are represented by ***, ** and * for *P *< 0.001, *P *< 0.01 and *P *< 0.05, respectively. Codes are as in table 1.

### DNA Analysis

All colonies found in either shallow or deep areas were classified as deep habitat or shallow habitat. Morphology was used to define the second group of populations (deep or shallow types). The two classification schemes are not identical, as the atypical colonies found during the morphological analysis could be correctly classified. Also, each reef was treated as one population so that among reef comparisons could be established.

#### Msh1

A total of 130 sequences of msh1 (723 bp) resulted in 10 distinct haplotypes, with three to nine haplotypes per population. The numbers of segregating sites were similar between populations, 9 and 8 sites were observed for colonies inhabiting shallow and deep environments, respectively.

The shallow type possessed 6 haplotypes and the deep type contained 4 haplotypes, among which the maximum difference was 6 substitutions. There were 2 and 3 segregating sites in the shallow and deep types, respectively. The most common haplotypes within each of shallow and deep types were represented by 54 and 30 individuals, respectively. The values of the nucleotide diversity indices (*π*, *θ*) for the pooled data were 0.0039 and 0.0024, respectively. *π *ranged from 0.0007 (shallow type) to 0.0039 (Romero reef), whereas *θ *was lowest in deep type (0.0009) and highest in Media Luna reef (0.0028). Most of the haplotypes were singletons; this mutation pattern was more common in colonies of shallow habitats. Fu's *Fs *test for the shallow and deep type revealed a significant departure from equilibrium only for the shallow type (-3.05038, *P *= 0.037). The excess of rare mutations observed in the shallow type is consistent with population expansion or purifying selection. Tajima's *D *tests were not significant.

The within shallow and deep type divergence varied from 0% to 0.3%, as estimated with the Kimura-2-parameter model [50]. Divergence between shallow and deep types ranged from 0.61% to 1.07%. The shallow type was more closely related (0.61% to 0.76%) to *Plexaura homomalla *than the deep type (0.92% to 1.23%).

#### 18S

A 251 bp fragment of 18S was sequenced from 143 colonies of *E. flexuosa*. Most of the sequences (90) were from Media Luna reef (colonies used in the transplant experiment), while 32 sequences were from Romero and an additional 21 from Culebra. Among all sequences, 15 haplotypes were discernable with colonies in shallow habitats presenting the highest haplotype diversity (h = 11). Among reefs, Media Luna had the highest number of haplotypes (h = 11). The values of the nucleotide diversity indices (*π*, *θ*) for the pooled data were 0.0088 and 0.0079, respectively. *π *ranged from 0.0014 (deep type) to 0.0093 (Romero reef), whereas the lowest (0.0044) and the highest *θ *(0.0089) was also observed in the same data partitions. The within shallow type genetic variability ranged from 0% to 0.97%, and the within deep type variability ranged from 0 to 0.48%, as estimated by the Kimura-2-parameter model. Divergence between shallow and deep types was similar to the *msh1 *divergence and ranged from 0.97% to 1.95%. Fu's *Fs *test for the data partitions revealed a significant departure from equilibrium for the shallow type (-3.7390, *P *= 0.0459) and the deep type (-4.4450, *P *= 0.0036). The excess of rare mutations observed in the ribosomal gene of the shallow type may be explained by population expansion or purifying selection. Tajima's *D *was significant only for the deep type (-1.6119, *P *= 0.0224). Departure from equilibrium was consistently observed across the two genes only for the shallow type group. The significant departure from neutrality recorded in the deep type by the 18S gene and not the *msh1 *gene, may be attributed to the fact that the two genes belong to different genomes and are under different selection and stochastic processes.

**Figure 5:**
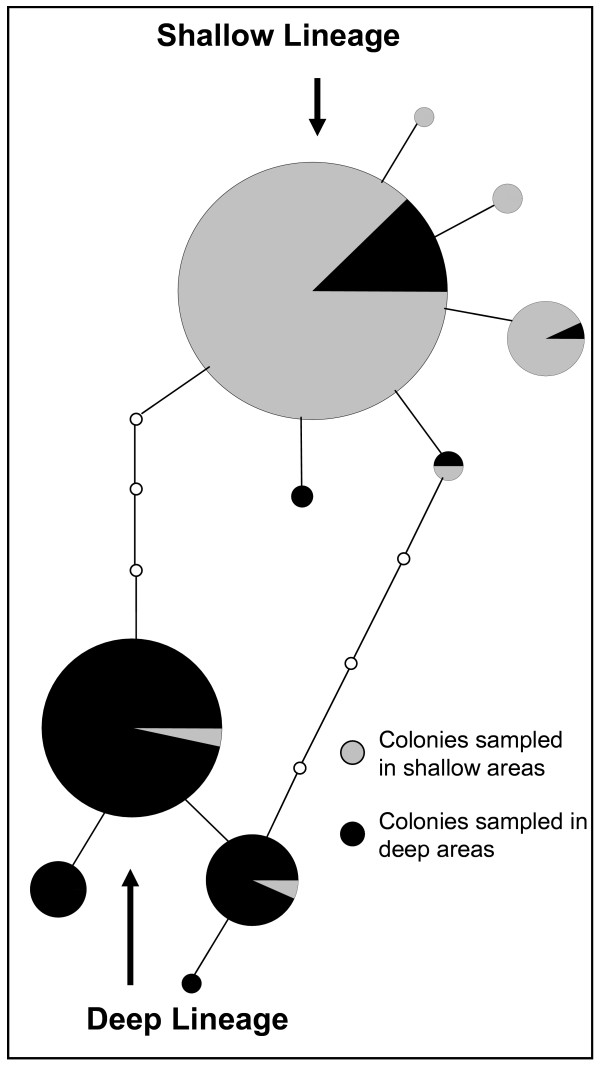
Parsimony haplotype networks based on *msh1 *for colonies inhabiting shallow or deep areas. The network indicates the six haplotypes found in shallow areas (i.e., shallow type) and the four haplotypes found in the deep areas (i.e., deeptype). The size of the circle is proportional to the observed number of sequences for the corresponding haplotype. The minimum number of steps is represented by the small empty circles.

AMOVA tests showed significant differentiation among populations (Table 4), regardless whether the populations were divided per habitat (shallow or deep) or by morphotype (shallow type and deep type). Nonetheless, population differentiation was maximized when the latter assignment was used. A comparison among reefs (Media Luna and Romero) yielded non-significant *F*_ST _values, suggesting an extensive gene flow among these two reefs. Also, a comparison of all shallow habitats (Romero shallow, Media Luna shallow and Culebra) suggested homogeneity among populations. The genetic homogeneity within La Parguera is not surprising because of the short distance between the reefs. Even when including colonies from Culebra, which is 100 km northeast from La Parguera, genetic homogeneity was still observed. However, when comparisons of reefs lacking the deep habitats (e.g., in Culebra only shallow areas were sampled), *F*_ST _values were significant, suggesting population subdivision. As inferred from the AMOVAs the haplotype network analysis showed similar patterns (Fig. 5). Two groups were extracted from the haplotype network analysis, the first consists of the vast majority of sequences that have the shallow morphotype and the second group contains most of the deep morphotype.

**Table 4:** Analysis of molecular variance (AMOVA) for *msh1 *and 18S (in parenthesis).

Comparison tested	d.f.	Sum of squares	Variance component	% variation	Φ_ST_
**Between habitats (shallow Vs deep)**					
Among populations	1 (1)	84. 89 (55.599)	1.29890 (0.79038)_Va_	66.87 (49.27)	0.66873 (0.49266)***
Within populations	128 (141)	82.36 (114.765)	0.64345 (0.81394)_Vb_	33.13 (50.73)	
Total	129 (142)	167.254 (170.364)	1.94235 (1.60431)		
**Between morphotypes (shallowtype Vs deeptype)**					
Among populations	1 (1)	130.412 (108.532)	2.06222 (1.59683)_Va_	87.89 (78.45)	0.87891 (0.78455)***
Within populations	128 (141)	36.084 (61.832)	0.28413 (0.43852)_Vb_	12.11 (21.55)	
Total	129 (142)	167.254 (170.364)	1.94235 (2.03536)		
**Between reefs (Romero Vs Media Luna)**					
Among populations	1 (1)	1.702 (0.642)	0.00829 (0.01242)_Va_	0.64 (0.96)	0.00637 (0.01043)
Within populations	128 (107)	165.552 (128.744)	1.29338 (1.20321)_Vb_	99.36 (99.04)	
Total	129 (108)	167.254 (129.385)	1.94235 (1.19079)		
**Between reefs considering shallow populations only (Romero Vs Media Luna Vs Culebra)**					
Among populations	2 (2)	0.921 (1.790)	0.00157 (0.00257)_Va_	0.37 (0.30)	0.00366 (0.00305)
Within populations	65 (74)	27.844 (62.210)	0.42837 (0.84067)_Vb_	99.63 (99.70)	
Total	67 (76)	167.254 (64.000)	1.94235 (0.84324)		
**Among reefs with different depth profiles (Media Luna Vs Romero Vs Culebra)**					
Among populations	2 (2)	15.034 (4.639)	0.18265 (0.03526)_Va_	13.22 (2.91)	0.13223 (0.02911)***
Within populations	127 (120)	152.22 (141.101)	1.19859 (1.17584)_Vb_	86.78 (97.09)	
Total	129 (122)	167.254 (145.740)	1.94235 (1.21110)		

Comparisons of population were made among depth profiles, morphotypes and reefs. The significance of the Φ_ST _values were obtained by randomizations of 10,000 permutations. *P *< 0.001 is represented by ***. Sampling in Culebra is limited to shallow habitats.

Gene genealogies were constructed in PAUP using ML with the HKY and Jukes Cantor as most suitable substitution models for *msh1 *and 18S, respectively. Analysis performed using neighbour joining and parsimony yielded similar patterns. Also, the topology of *msh1 *and 18S was similar, however the 18S analysis recovered only one of the clades, as 18S was less variable than *msh1*.

**Figure 6:**
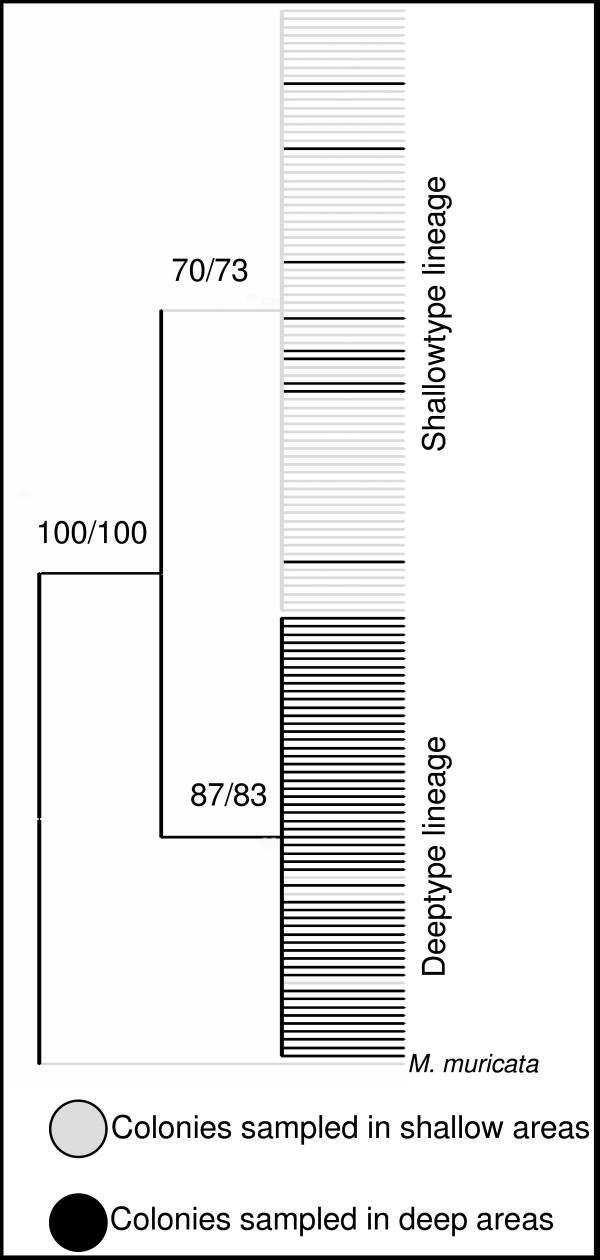
Gene genealogy for *msh1*. Bootstrap values for 100 and 1000 replicates in maximum likelihood and neighbor joining analyses are shown. Similar topologies and bootstrap values were recovered using maximum parsimony but not shown. *Muricea muricata *was used as an outgroup. In grey are colonies sampled in shallow areas and in black colonies in deep areas. The genealogy divided the individuals in two clades, which are highly indicative of the habitat origin (shallow or deep). In one clade (bottom) most of the colonies (62) are from deep areas (black), however 9 colonies (grey) fall within this clade. These 9 colonies had a different morphology despite living in the same habitat. The same pattern was observed for the top clade.

The genealogy divided the individuals in two clades (Fig. 6), which are highly indicative of the habitat origin (shallow or deep). In the bottom clade, 62 of the colonies are from deep areas; however nine colonies fell within this clade. These nine colonies are the atypical colonies found during the morphological analysis (e.g., Media Luna shallow), where comparisons of morphological characters of these atypical colonies did not match the overall population mean, despite living in the same habitat. Similar patterns are displayed for the top clade, which represents 68 colonies sampled in shallow areas and three atypical colonies found in deep areas. Furthermore, these atypical colonies in each clade are those colonies that were misclassified (7%) by the discriminant function analysis. Table 5 shows a summary of the number of individuals within each genetic lineage divided by habitat and morphotype (shallow and deep types). The atypical colonies within each habitat were later shown by the DNA analysis (Fig. 6) to be phylogenetically closer to their opposite habitat congeners, reinforcing the morphological differences.

**Table 5:** Number of individuals in each genetic lineage.

	Habitat (shallow and deep)	
**Genetic lineage**	**Shallow**	**Deep**

**G1**	65	3
**G2**	9	53
	**Morphotypes (shallow-type and deep-type)**	
**Genetic lineage**	**Shallow-type**	**Deep-type**
**G1**	68	0
**G2**	0	62

In the top-half of the table the genetic lineages are divided by their living habitat. The bottom-half shows the genetic lineage division based on morphology (morphotypes).

## Discussion

*Eunicea flexuosa *is divided into two discrete morphological forms in southwest Puerto Rico. The shallow type is pervasive in shallow areas, but a few colonies of the shallow type can be found in deep habitats. The second morphotype (deep type) could be described as small colonies, with fewer terminal branches, more sparsely polyps, thinner branches and bigger spindles. The deep type is found at the muddy bed at the base of forereef of inside and mid reefs and largely confined to deep areas with low water motion patterns, high sediment transport and lower light levels. The observed variation in *E. flexuosa *resembles findings on phenotypic plasticity of other modular organisms. Mechanical stimuli such as wind speed or water motion induce morphological adjustments in both terrestrial and marine plants [4,51]. Other marine modular taxa such as the bryozoan *Membranipora membranacea *adapt to water flow to maximize food capture [52], the demosponge *Halichondria panacea *develop stiffer branches in high energy habitats [5] and the coral *Madracis mirabilis *exhibit sparser and thicker branches in high flow areas [10]. Light has also been associated with phenotypic changes in plants [53], algae [54] and corals [55]. Other non-mechanical stimuli such as presence of predators in the bryozoan *M. membranacea *can induce plastic responses (i.e. defensive spines) [9].

The discrete morphological distribution found in *E. flexuosa *is in concordance with previous studies of octocorals, where colonies inhabiting deep forereef areas were thinner with more sparse and fewer calices and have bigger spindles than the colonies in shallow habitats in either back or forereef areas in the Florida Keys [12]. Spindles at the branch tip were significantly smaller and presumably underdeveloped in *E. flexuosa*, while in *Briareum asbestinum*, West [47] reported bigger spindles at the tips. The discrepancy of the findings may be related to different ecological pressures on the two species, different function of spindles in each species (i.e., *B. asbestinum *lacks a central axis) or may depict difference responses constrained by their separate phylogenetic history. Moreover in terms of habitat differences, smaller spindles at high water motion areas are thought to increase stiffness of the colony to avoid breakage. Grigg [56], West et al. [14] and Kim et al. [12] reported that sclerite size is negatively correlated with water motion for *B. asbestinum *and *E. flexuosa*. However, the opposite observation has been made in *Eunicella singularis *[45]. Colony size, calice diameter and polyp density may compensate water flow changes and enhance respiration, feeding or structure as has been reported for scleractinian corals [10,57,58]. Furthermore, the overall discrepancy in colony development (more and bigger terminal branches) may be interpreted as a response to the water drag forces acting upon the colony, yet colonies in shallow areas are exposed to higher water flow and vice versa.

In *E. flexuosa*, the differences of the two morphotypes are related to depth profiles, water motion and sediment transport. However, as water motion, sediment transport and light co-vary along depth profiles, the study could not distinguish the individual influences. Furthermore, the deep habitats of the inside and mid reefs differ significantly from those of the outer reefs. In the inside and mid reefs, loose sediments move along the slope and are deposited at the base of the forereef, where deep colonies were sampled. Therefore, settling larvae in such areas are subject to higher sediment deposition and probably have developed an adaptational response to such environmental condition. Similarly, colonies in the two outer reefs, El Hoyo and Weinberg, developed in the edges of sand patches were different than those spread along the hard ground bottom. In La Parguera, the octocoral community observed at the sand bed adjacent to the forereef (~20 m), is less diverse than the community of hard ground habitats. In such suboptimal habitats, *Pseudopterogorgia *spp., *Plexaurella *spp. and some *Muricea *spp. (except *Muricia muricata*) are the dominant species. These species are probably better adapted to overcome sediment suffocation and develop. *Pseudopterogorgia *spp. and *Muricea *spp. avoid sediment burial by rapidly reaching a safe size due to the lack of thick central axis which allows for faster growth. In *Plexaurella *spp., the presence of big polyps can efficiently remove sediment from the colony. The deep type of *E. flexuosa *is a thin colony with a small central axis and few polyps, which might grow fast enough to overcome sediment overload.

The morphology of *E. flexuosa *can also be affected by light and other factors associated with depth such as food resources, presence of predators and hydrostatic pressure. Nonetheless, water motion covaries with depth and therefore both are related. Light is likely a major factor related to depth and affects anthozoan morphology, as presence of zooxanthellae is tightly related to carbonate uptake and energy supply for the host. Light levels influence the phenotype of anthozoans due to changes in zooxanthellae concentration and decrease of calcification rates [14,55,59,60]. Moreover, plate-like colonies most likely represent an evolutionary response to compensate for low levels of light by increasing area and zooxanthellae concentrations.

The two morphologies of *E. flexuosa *associated with different habitats showed some degree of phenotypic plasticity in sclerite characters (especially spindles), which showed a clear tendency to increase and decrease when transplanted to deep and shallow areas, respectively. Nonetheless, after the 15 months of the experiment in neither case the spindles of transplanted colonies became similar in size as the residents' spindles. The results suggest that either there was not enough time for the colonies to produce new tissue under the novel conditions or there is a non-environmental factor accounting for the rest of the morphological variation. There is less evidence for the former; colonies grew on average ~2 cm during the transplant experiment, and since the analysis was performed using tissue 1 cm from the branch tip, there was more than 0.5 cm tissue to be analyzed. Also, the reaction norm graphs showed a stabilization of the curve at 10 and 15 months, suggesting that the maximum of variation was reached.

Gorgonian corals resemble plants in many ways and morphologically they are modular organisms where integration/disintegration can occur in response to environmental gradients [61]. If phenotypic plasticity in gorgonians is expressed at the modular level (polyp and branch), the transplant results may be interpreted as the sum of all historical (branches before transplantation) and experimental (branches during transplantation) interactive and antagonistic changes between modules. The observed changes in sclerite size which are changes affecting individual modules suggest there is some plasticity at the modular level. The plastic response of modules has cascade effects on the colony level. For example, when colonies are transplanted from deep to shallow areas, sclerites become smaller and denser enhancing the flexibility of the colony.

Other transplant experiments with modular organisms (e.g., the sponge *Halichondria panacea*) exhibit variable responses to environmental stimuli [5]. When colonies of *H. panacea *were placed in high energy areas, they became stiffer, a response which is in agreement with our observations on *E. flexuosa*. Alternatively, plasticity may be most likely promoted during the early life stages rather than in mature colonies. Other stochastic environmental factors such as the 2005 Caribbean wide bleaching event may have affected the results.

The possibility that no environmental factors account for the majority of morphological variation is supported by the genetic data. Analysis of the *msh1 *and 18S genes resulted in two major lineages associated with depth. Colonies transplanted from shallow to deep habitats suffered about 50% mortality; those transplanted from deep to shallow suffered 20% mortality. Since survivorship is a fundamental attribute of fitness then increase in mortality would indicate lower fitness. Therefore, each morphotype is better adapted to the deep or shallow areas. The results from the transplant experiments may represent experimental error or a drastic response of the colonies to novel environmental conditions. Previous studies have shown that adult colonies (> 20 cm) in natural populations are stable and have a normal survivorship rates above 90% [62], suggesting that such high mortalities are natural responses rather than sampling error. Although the two genetic lineages are associated with the two habitats, there is a noticeable response of both shallow and deep type to environmental stimuli. As previous studies have reported, there is a positive correlation of spindles to increase in size as depth increases and vice versa [12,14]. Clubs and capstans slightly changed with depth but the tendency was not consistent.

In Puerto Rico, the morphological divergence found in colonies of *E. flexuosa *is genetically based. Gene genealogies, haplotype networks and AMOVA analysis of both nuclear and mitochondrial genes suggested that such discrete morphological distribution is correlated with the presence of two distinct lineages, distributed non-randomly in shallow and deep environments. However, either of the two lineages can infrequently be found in both depth habitats. The genetic break found in nuclear and mitochondrial DNA suggests that gene flow ceased a long time ago and divergence may have led to speciation. Fixed differences in both nuclear and mitochondrial genes are comparable to those reported between species of octocorals [63,64]. Therefore, the current description *sensu *Bayer [46] of *E. flexuosa *is a complex of at least two distinct genetic lineages, adapted to different habitats and that do not exchange genetic material despite living in sympatry. The extensive distribution and ample morphological variation corresponds to two distinct genetic lineages with narrower distributions and more rigid phenotypic plasticity. The observed genetic pattern may have resulted from 1) secondary contact after populations diverged in allopatry and reproductive incompatibility developed, 2) by divergence with gene flow through ecological specialization in sympatry or 3) by the poorly understood process of hybridization in anthozoan evolution.

### Processes of Divergence

The planulae of most anthozoan broadcasters such as *E. flexuosa *are capable of staying days to weeks in the water column producing genetically homogeneous populations across large geographic areas [21,22,65]. Despite the high potential for dispersion and population connectivity, allopatric speciation is probably the most common mode of speciation in marine environments [23,24,66,67]. Recent genetic studies [68-71] have contradicted earlier assumptions of population homogeneity in Caribbean populations of marine taxa [72,73]. Even though allopatric distribution could have caused the divergence of the lineages, there is no recent evidence of geological processes that may have altered the patterns of Caribbean circulation. However, distinct sympatric lineages have been uncovered in other Caribbean invertebrates [74].

On the other hand, sympatric speciation by ecological differentiation [17] and disruption of gene flow in proximate populations is plausible. The two genetic lineages of *E. flexuosa *are found at the opposite ends of a depth gradient. Diversifying selection may favor the two phenotypes at the extremes of the depth gradient, preventing gene flow through assortative mating and eventually leading to new species [75]. These ecological specializations to depth habitats have been pointed out in earlier reviews [32]. Such ecological differences in niche utilization may be reinforced by dissimilar characteristics associated with the habitats they occupy. Symbiotic relationships to host [76] or to environment [77], differential timing of gamete release due to depth related differences [34,35] may have provided different resources to populations at different habitats and eventually prevent random mating. As a consequence, rapid evolution of mating systems may have been favoured [37-39]. In Caribbean corals, diversifying selection has been proposed in at least two species: *Montastraea annularis *[27] and *Favia fragum *[30]. It is likely that the genetic differences reported by Brazeau and Harvell [29] in the gorgonian *B. asbestinum *could have arisen through the same mechanism. Although, disruptive selection seems to explain the divergence of the two lineages of *E. flexuosa*, hybridization is also another plausible mechanism to cause diversification in marine taxa.

Hybridization is a common phenomenon in plants and the rise of new lineages due to reticulations is often reported in flowering plants [41,78]. In the marine environment, there are instances of hybridization in angelfishes [79], cichlids [80], blue mussels [81] and corals [82-84], suggesting that hybridization is an important evolutionary mechanism for speciation. Nonetheless, it is often assumed that discrepancies in gene phylogenies or *F*_ST _statistics from different molecular markers is interpreted as incomplete lineage sorting, rather than reticulations as it is interpreted often in plants [41,78]. The phenomenon of reticulate evolution may have great influence in the speciation of marine species especially those living in sympatry with high potential for hybridization (e.g., spawners).

Veron [43] has provided a theoretical framework to consider reticulate evolution as an important factor of coral evolution. Direct measures of chromosome differences established in *Acropora *[85], genetic surveys of the nuclear genome of corals [83,84,86] and direct crosses of gametes [82] have shown introgression in natural populations. In *E. flexuosa*, the two uncovered lineages may have arisen by hybridization between the common form of *E. flexuosa *with another *Eunicea or Plexaura*species. Furthermore, hybrid fitness may increase over parent fitness in novel environment or in extreme habitats. Hybrids tend to explore novel habitats avoiding introgression and competition with their parents [87,88]. Therefore, it is likely that the lineage related to deep muddy areas is of hybrid origin and is better adapted to such conditions than the parental species.

## Conclusion

The accepted description *sensu *Bayer [46] of *E. flexuosa *is a complex of at least two distinct genetic lineages, adapted to different habitats and that do not exchange genetic material despite living in sympatry. The present study highlights the importance of correctly defining species, because the unknowingly use of species complexes can overestimate geographical distribution, population abundance, and physiological tolerance. The non-random distribution of both morphotypes can yield misleading population genetic inferences in the absence of adequate taxonomy. Consequently, decisions based on these estimates will have repercussions in conservation programs [32]. Detailed studies of the mechanism by which anthozoans achieve assortative mating and become reproductively isolated would give us insights in the speciation process. Also, cross fertilization experiments, genetic assessment of shared alleles through genetic markers and karyotyping may shed light on the speciation process via hybridization. Reticulations are common in plants, a group that resembles most of the ecological aspects (bet hedging strategies, modular organization, philopatric recruitment, etc.) that govern marine modular organisms.

## Methods

### The Species

Formerly known as *Plexaura flexuosa*, the species has been recently placed into the genus *Eunicea *based on molecular data and the size ratio of spindles and clubs [89]. *Eunicea flexuosa *is an octocoral cnidarian forming colonies of ~1 m in height found in coral reefs and tropical rocky walls [46,49,90]. *Eunicea flexuosa *is relatively abundant in low relief hard ground habitats with preference for high water motion areas [48,49]. The distribution of the species spans through several environmental gradients (e.g., depth, light, water motion and sediment transport) [90] and display an unusual amount of morphological variability. *Eunicea flexuosa *is a gonochoric gorgonian that reproduces sexually by spawning gametes [91]. Asexual reproduction through fragmentation can also take place when loose branches spread to the surroundings (< 10 m) and new colonies develop from clone-mate propagules but is not as common as in *P. kuna *[92]. The colony is produced by the asexual budding of its polyps, generating a branching dichotomous morphology, arranged in an axial skeleton of fused sclerites of calcium carbonate [46]. Adult colonies exhibited bush-like shape and branch profusely in a single plane. Microscopically, the apertures (calices) present an inconspicuous lower lip with an unarmed collaret. Sclerites are arranged in three layers. The axial sheath is made up of fused capstans usually purple and ≤ 200 μm in length. The external layer contains leaf clubs of ~200 μm with 3 or 4 serrate folia and structural spindles (~2000 μm in length) are disposed in a mid layer [46]. The last two features distinguish the species from the other plexaurids. For both *P. homomalla *and *P. nina *the leaf clubs and spindles are smaller (150 μm and 700 μm, respectively), spindles are also more slender and calyces usually exerted in *P. nina *(Bayer 1961). Contrary to *P. homomalla*, colonies of *E. flexuosa *in the field usually tend to be branched in one plane but not in a net-like shape with calyces as a lower lip. All colonies sampled at different depths and reefs fell within the original description of *Eunicea flexuosa *(*sensu *Bayer 1961, modified by Grajales et al. 2007). None of the colonies exhibited prominent and well developed calyces, typical of other *Eunicea *species.

### The Environment

The study was carried out in La Parguera southwest Puerto Rico (Table 6) during September 2004 to August 2006. The hydrography of the area has been studied and described elsewhere [93]. Reefs off La Parguera are exposed to wave action, generated by the easterly trade winds. The shallow (< 10 m) benthic community is visually dominated by octocorals, forming colourful gardens, especially in shallow reef platforms with high water motion and wave energy. The current abundance of gorgonians may have been enhanced by the die off of the herbivorous sea urchin *Diadema antillarum *[62]. La Parguera is composed by three prominent reef formations, located parallel to the shoreline: 1) inshore reefs which are more protected to waves and currents, but subjected to higher sedimentation rates, direct contact with sewage discharges and lower scleractinian cover, 2) mid reefs and 3) outer reefs which are fully exposed to wave energy, with higher coral cover and significant bottom relief.

**Table 6:** Relative differences in environmental characteristics of the sampling locations.

Locations	Coordinates	Zone	Depth (m)	Depth	Water Flow (cm/s)	Sedimentation rates (g/day)
**Pelotas**	17°57.442 N – 67°04.176 W	Inner	3	PS	4	0.28 (0.18)
			17	PD	9.3	0.11 (0.05)
**Conservas**	17°57.336 N – 67°02.569 W		3	CS	5.6	*
			17	CD	9.5	*
**Romero**	17°56.249 N – 66°59.443W		3	RS	> 19.5	0.86 (0.69)
			18	RD	< 13.5	0.34 (0.25)
**Turrumote**	17°56.097 N – 67°01.130 W	Mid	3	TS	> 25.5	0.46 (0.21)
			17	TD	<14.0	0.15 (0.11)
**Media Luna**	17°56.093 N – 67°02.931 W		3	MS	> 25.5	0.32 (0.3)
			20	MD	< 14.3	0.15 (0.14)
**El Hoyo**	17°52.559 N – 67°02.619 W	Outer	22	H	>23.3	0.09 (0.11)
**Weinberg**	17°53.429 N – 66°59.320 W		23	W	>23.3	0.04 (0.03)

Estimations on water flow were taken from McGehee [113]; standard deviations for the data were not available. Sedimentation rates are reported as mean values ± standard deviation (Coral Reef Ecosystem Studies Project, unpublished data). * Represents not available data for that reef, however the sedimentation patterns are similar to those in Pelotas.

Gorgonian colonies were sampled in seven reefs for morphological measurements (Table 6). Three inshore, protected reefs (Conservas, Pelotas and Romeo); two mid-shelf reefs exposed to wave action (Media Luna and Turrumote) and two outer-shelf reefs (El Hoyo and Weinberg) were included. In each site, morphological variability of *E. flexuosa *was assessed at two depths (shallow < 5 m and deep > 17 m), except in the outer reefs El Hoyo and Weinberg, which are at depth 23–27 m. Therefore, in most reefs there are two depths, except the outer reefs. In each location, 15 colonies were sampled (n = 30/reef).

### Natural Variability of Morphological Traits

**Figure 7:**
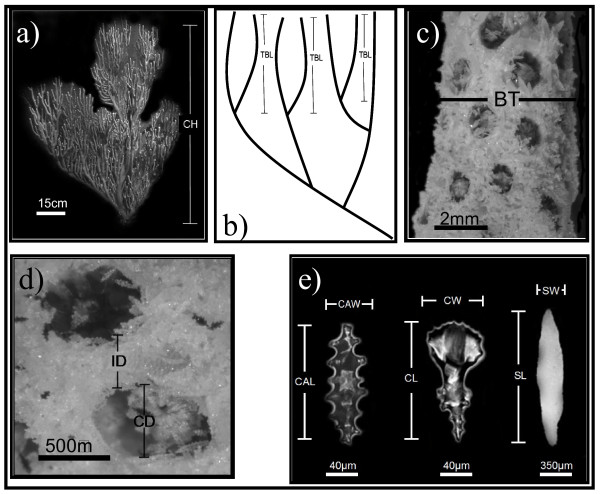
Phenotypic traits measured in *E. flexuosa*. a) colony height (CH); b) average length of terminal branches (TBL) and number of terminal branches (TBN); c) branch thickness (BT); d) calice diameter (CD) and intercalice distance (ID) and e) length and width of capstans (CAL and CAW), clubs (CL and CW) and spindles (SL and SW). Surface area (SA) and polyp density (PD) were measured in 1 linear cm.

Three macro-morphological traits: colony height (CH), branch thickness (BT) and branch development (BD) were measured in 180 colonies, representing fifteen colonies per location (7 reefs per 2 depths, except El Hoyo and Weinberg which represent deep habitats). Branch development was assessed as the total number of terminal branches (Fig. 7b) and the average length of 10 haphazardly chosen branches from branch tip to the first node in terminal branches (Fig. 7b).

Nine microscopic characters from the same 180 colonies were measured at 2 cm off the tip to avoid measuring underdeveloped sclerites because new tissue is generated at the tips and contains higher number of smaller sclerites, especially in the spindle width and length [94]. Two cm off the branch tip was far enough to avoid smaller, underdeveloped sclerites, but within the range in which the new tissue would be generated under the novel environmental conditions during the reciprocal transplant experiment. Polyp density (PD), calice diameter (CD), inter-calice distance (ID) and width and length of external clubs (CW and CL) mid-layer spindles (SW and SL) and axial sheath capstans (CAW and CAL) were selected for the analysis (Fig. 7). Polyp density was estimated by counting the number of polyps/cm^2 ^and standardized with branch thickness, assuming cylindrical shape of the branches. In these micro-morphological traits, 20 measurements (except polyp density) were performed in randomly selected calices in CD and ID or sclerites in CW, CL, SW, SL, CAW and CAL; representing 300 measurements per depth, 600 per reef, 3,600 per character and 28,800 in total, excluding polyp density measures. Octocoral branches were collected by clipping off a 5 cm section at the branch tip, slightly bleached with Clorox (5%) to remove some tissue, rinsed in distilled water, and dried. The slight bleaching kept the colony shape intact, avoiding colony dissolution while allowing clear observations of the calices. For sclerite analysis 1 cm section at 2 cm from the tip was collected and dissolved with Clorox (5%), following Bayer's protocol [46]. For clubs and capstans the samples were taken by placing the spicules in slides and random samples were obtained by moving blindly the slide and measuring all sclerites in each new visual field until 20 sclerites were measured. Spindle, calice and polyp were analyzed by photographing the characters with an Olympus BX-51 compound microscope. All measurements were carried out using photographs (calibrated with a slide of 10 μm accuracy) taken with an Olympus C-5050 camera system attached to an Olympus SZH-10 stereo microscope. Analysis of the photos was performed using SigmaScan (SPSS Inc.).

The extent of variation among habitats (i.e., PS, PD) in morphological traits (14) was analyzed by individual One-way ANOVAs. Principal component analysis (PCA) was applied to test the association of the traits and visually examine overall trends, the analysis was done with both raw and standardized (in a 0 to 1 scale) data. The PCA scores of the first three principal components were used to test for differences among groups and factor interactions in a two-way ANOVA. The two factors were degree of protection (zone) and depth (< 5 m and < 17 m). Reefs were nested within zones and depth was factorial with reefs. Kolmogorov-Smirnov and Levene's test were used to test for normality and homogeneity of variance, respectively [95]. Discriminant function analysis (DFA) of 14 morphological characters (CH, BT, BD, PD, CD, ID, CW, CL, SW, SL, CAW and CAL, Fig. 7) was used to explore if colonies could be assigned independently to one of the two resulting morphotypes and replicate the same predetermined groups in the sampled populations (i.e., inner zone, mid zone, shallow and deep). Wilk's λ was used in the DFA to test for multivariate differences among groups [96]. Individual DFA's were employed for depth, location and zone.

### Transplant Experiment

If reciprocally transplanted colonies become similar in sclerite size to the neighbouring colonies, the environment is likely to have an effect on the phenotype. As gorgonian growth is slow (< 3 cm a year; Yoshioka unpub. data) only sclerite related traits were evaluated. We examined the subapical parts of branches of transplanted gorgonians because this is where new spicules and skeleton material are generated. By avoiding the sampling of tips no underdeveloped sclerites were counted [94].

To test for eco-phenotypic responses of the species to different depth habitats, thirty branches (< 30 cm) of different colonies separated by >10 m from each other, from each depth were reciprocally transplanted in Media Luna reef. Additionally, 15 branches from each of the two depths were auto-transplanted (transplanted to the same depth) to serve as controls. In each depth, 45 branches were transplanted per depth. Fifteen of them were residents and 30 were introduced from either the shallow or the deep habitat, for a total of 90 colonies in the experiment. Each branch was glued into a short piece of plastic PVC pipe with marine hydraulic cement. The cement did not affect the octocoral growth, as live tissue quickly overgrew the dried cement; the same material has been successfully used in restoration of gorgonian populations (Yoshioka et al. unpub. data). Each of the plastic pipes was attached to a 1.5 × 1 m cement panel. Fifteen branches were randomly assigned in each cement panel (allowing enough distance to avoid conspecific aggressions) and three panels were placed per depth (45 colonies per depth) separated by 5 m from each other. Each branch was assigned a code and individually tagged on the panels. The same code was also used for genetic analysis. The experiments were monitored every month to remove fouling organisms and sediment from the panels. Three times during the 15 months of the study (once every five months) surveys were carried out and linear growth and survivorship recorded. As the transplanted colonies were introduced to the novel habitat, the survivorship and linear growth were used to evaluate the overall colony response in each treatment. Linear growth was measured from base to tip of the colony and survivorship was recorded as live or dead. Branch thickness and polyp density could also reflect phenotypic plasticity but these parameters were not measured because detectable variation requires a longer experimental time due to the slow growth (~3 cm y^-1^) of *E. flexuosa*.

Linear growth of the transplanted colonies was assessed by subtracting the initial length from the final length. A two-way ANOVA was performed to test for differences in population (colonies from shallow and deep areas) and depth (colonies grew in shallow and deep environments) and their interactions. Both factors were considered fixed effects. Chi-square analysis on the number of dead colonies was used to test for survivorship independence among groups. To check for environmental effects on the groups, morphological trends were visually examined by graphing the reaction norm through time per group per trait. Repetitive measurement ANOVAs were used to test the effect of population and time and their interaction. ANOVA assumptions were evaluated as previously described. All statistical analyses were performed in JMP ver 5.0.1, InfoStat ver. 2004, and SigmaStat.

### Genetic Analysis

Portions of *msh1 *and the 18S genes were sequenced to test for genetic differentiation among populations of *E. flexuosa *living in different depths and reefs (Media Luna, Romero and Isla Culebra) in Puerto Rico. *Msh1 *is a mitochondrial gene unique to octocorals that codes for a DNA mismatch repair protein and is not present in other Cnidaria [97]. *Msh1 *and the nuclear 18S provide enough resolution to discriminate between closely related species of octocorals [63,64]. Segments of *msh1 *and 18S genes of all transplanted colonies in the eco-phenotypic experiment were examined, 90 colonies in total (45 individuals for each depth habitat). Additional samples from Romero and Culebra were included in the analysis to test the reproducibility of the preliminary results found in Media Luna. Thirty one colonies from Romero and 21 colonies from Culebra were included for the *msh1 *analysis. Thirty two colonies from Romero and 21 from Culebra were included for the 18S analysis. A 3 to 5 cm colony fragment was brought to the laboratory for immediate DNA extraction or stored in 95% ethanol for subsequent work. The PureGene DNA isolation kit (Gentra) was used for DNA extraction. The PCR amplifications was performed in an Eppendorf MasterCycler with the same cycling conditions for both genes, consisted of an initial denaturation at 95°C for 3 min, followed by a touch-down routine of annealing of 10 cycles at 43°C of 45 sec and 25 cycles at 48°C of 45 sec; denaturation at 94°C for 45 sec and elongation at 72°C for 5 min. The primers used to amplify 18S (A18S – 5'-GATCGAACGGTTTAGTGAGG-3' and ITS-4 – 5'-TCCTCCGCTTATTGATATGC-3') and *msh1 *were developed by Takabayashi [98] and France and Hoover [99], respectively. Sequencing reactions were prepared with a DYEnamic ET Terminator Cycle Sequencing Kit (GE) and loaded in a MEGABase 96 lane Sequencer for capillary electrophoresis.

DNA sequencing trace files were imported into the Phrap/Phred/Consed programs [100] for base calling, quality assessment, assembly and visualization. Mutations were verified in both the forward and reverse direction. Sequences were then aligned in MacClade [101] and compared with BLAST to publicly available sequences of closely related gorgonians. The haplotype (h) and the nucleotide diversity (*π*), number of segregating sites (*S*), the Watterson's estimator (*θ*_*w*_) were evaluated according to Nei [102] as implemented in DNAsp 4.0 [103]. Tajima's *D *[104] and Fu's *Fs *[105] were used in ARLEQUIN [106] to test for deviations from neutrality. A parsimony haplotype network was constructed for the *msh1 *sequences using the Templeton et al. [107] algorithm as implemented in TCS version 1.21 [108]. The parsimony network was constructed with confidence level set at 95%. Analysis of molecular variation [AMOVA, [109]] of among reefs, between habitats and between morphotypes was performed in ARLEQUIN. The AMOVAs were performed with 10,000 permutations by using conventional *F*-statistics with haplotype frequencies.

Gene genealogies were constructed for *msh1 *and 18S using the maximum likelihood (ML) method in PAUP* [110]. Hierarchical likelihood ratio tests in MODELTEST 3.6 [111] suggested that the HKY [112] and JC models were the best substitution models for *msh1 *and 18S, respectively. For the heuristic searches in ML, data were bootstrapped 100 times, and sequences were added randomly ten times. Phylogenetic relationships were also constructed using neighbor-joining and maximum parsimony with 1000 bootstrap replicates as implemented in PAUP*. Given the uncertain phylogenetic position of *E. flexuosa *and its conspecifics [64], several outgroups were used, including *P. homomalla*, *Muricea muricata *and *Eunicea *spp. Regardless the outgroup, identical topologies were obtained. All sequences have been deposited in GenBank (Accession numbers EF659469-EF659598 and EF659599- EF659741)

## Authors' contributions

CP conceived the project, collected the data, carried out the analyses and wrote the manuscript. PY and NVS advised on analyses, NVS provided samples and lab materials. PY and NVS participated in discussions. All authors read and approved the final manuscript.

## References

[B1] Pigliucci M Evolution of phenotypic plasticity: where are we going now? Trends Ecol Evol 2005 20 9 481 486 10.1016/j.tree.2005.06.001 16701424

[B2] Futuyma DJ Evolutionary Biology 1998 Sunderland, MA. , Sinauer

[B3] Schlichting CD The evolution of phenotypic plasticity in plants Annu Rev Ecol Syst 1986 17 667 693 10.1146/annurev.es.17.110186.003315

[B4] Fowler-Walker MJ Wernberg T Connell SD Differences in kelp morphology between wave sheltered and exposed localities: morphologically plastic or fixed traits? Mar Biol 2006 148 755 767 10.1007/s00227-005-0125-z

[B5] Palumbi SR Tactics of acclimation: morphological changes of sponges in an unpredictable environment Science 1984 225 1478 1480 10.1126/science.225.4669.1478 17770077

[B6] Marchinko KB Dramatic phenotypic plasticity in barnacle legs (Balanus glandula Darwin): Magnitude, age-dependence and speed of response Evolution 2003 57 1281 1290 12894936 10.1111/j.0014-3820.2003.tb00336.x

[B7] Trussell GC Phenotypic plasticity in an intertidal snail: the role of a common crab predator Evolution 1996 50 448 454 10.2307/2410815 28568849

[B8] Vermeij GJ Phenotypic evolution in a poorly dispersing snail after arrival of a predator Nature 1982 299 349 350 10.1038/299349a0

[B9] Yoshioka PM Predator-Induced Polymorphism in the Bryozoan Membranipora membranacea J Exp Mar Biol Ecol 1982 61 233 242 10.1016/0022-0981(82)90071-5

[B10] Bruno JF Edmunds PJ Clonal variation for phenotypic plasticity in the coral Madracis mirabilis Ecology 1997 78 2177 2190

[B11] Gleason DF The adaptive significance of morphological plasticity in the reef coral Porites astreoides Am Zool 1992 32 92D

[B12] Kim E Lasker HR Coffroth MA Kim K Morphological and genetic variation across reef habitats in a broadcast-spawning octocoral Hydrobiologia 2004 530/531 423–432 10.1007/s10750-004-2646-8

[B13] Takabayashi M Hoegh-Guldberg O Ecological and physiological differences between two colour morphs of the coral Pocillopora damicornis Mar Biol 1995 123 705 714 10.1007/BF00349113

[B14] West JM Harvell CD Walls AM Morphological plasticity in a gorgonian coral (Briareum asbestinum) over a depth cline Mar Ecol Prog Ser 1993 94 61 69 10.3354/meps094061

[B15] Willis BL Ayre DJ Asexual reproduction and genetic determination of growth form in the coral Pavona cactus: biochemical genetic and immunogenetic evidence Oecologia 1985 65 516 525 10.1007/BF00379666 28311859

[B16] Grajales A Aguilar C Sánchez JA Phylogenetic reconstruction using secondary structures of Internal Transcribed Spacer 2 (ITS2, rDNA): finding the molecular and morphological gap in Caribbean gorgonian corals BMC Evolutionary Biology 2007 7 90 10.1186/1471-2148-7-90 17562014 PMC1913914

[B17] Doebeli M Dieckmann U Speciation along environmental gradients Nature 2003 421 259 264 10.1038/nature01274 12529641

[B18] Hilbish TJ Demographic and temporal structure of an allele frequency cline in the mussel Mytilus edulis Mar Biol 1985 86 163 171 10.1007/BF00399023

[B19] Doebeli M Dieckmann U Metz JAJ Tautz D What we have also learned: adaptive speciation is theoretically plausible Evolution 2005 59 691 695 15856711

[B20] Grosberg RK Cunningham CW M. D. Bertness, S. Gaines, Hay ME Genetic structure in the sea: from populations to communities Marine Community Ecology 2001 Sunderland, MA , Sinauer 61 84

[B21] McFadden CS R. K. Grosberg B. B. Cameron D. P. Karlton D. Secord Genetic relationships within and between solitary and clonal forms of the sea anemone Anthopleura elegantissima revisited: Evidence for the existence of two species Mar Biol 1997 128 127 139 10.1007/s002270050076

[B22] Lessios HA Robertson DR Crossing the impassable: genetic connections in 20 reef fishes across the Eastern Pacific Barrier Proc R Soc Lond B 2006 273 2201 2208 10.1098/rspb.2006.3543 PMC1635520 16901840

[B23] Knowlton N Mills DEK The systematic importance of color and color Pattern: Evidence for complex of sibling species of snapping shrimp (Caridea: Alpheidae: Alpheus) from the Caribbean and pacific coasts of Panama Proc San Diego Soc Nat Hist 1992 18 1 5

[B24] Barber PH S. R. Palumbi M. V. Erdmann Moosa MK Sharp genetic breaks among populations of Haptosquilla pulchella (Stomatopoda) indicate limits to larval transport: patterns, causes, and consequences 2002 11 659 674 10.1046/j.1365-294x.2002.01468.x 11972755

[B25] Jiggins CD Mallet J Bimodal hybrid zones and speciation Trends Ecol Evol 2000 15 250 255 10.1016/S0169-5347(00)01873-5 10802556

[B26] Knowlton N Weil E Weigt LA Guzman HM Sibling species in Montastraea annularis, coral bleaching, and the coral climate record Science 1992 255 330 333 10.1126/science.255.5042.330 17779583

[B27] Weil E Knowlton N A multi-character analysis of the Caribbean coral Montastraea annularis (Ellis and Solander, 1786) and its two sibling species, M. faveolata (Ellis and Solander, 1786) and M. franksi (Gregory, 1895) Bull Mar Sci 1994 55 151 175

[B28] Palumbi SR Metz E Strong reproductive isolation in closely related tropical sea urchins (genus Echinometra) Mol Biol Evol 1991 8 227 239 2046543 10.1093/oxfordjournals.molbev.a040642

[B29] Brazeau DA Harvell CD Genetic structure of local populations and divergence between growth forms in a clonal invertebrate, the Caribbean octocoral Briareum asbestinum Mar Biol 1994 119 53 60 10.1007/BF00350106

[B30] Carlon DB Budd AF Incipient speciation across a depth gradient in a scleractinian coral? Evolution 2002 56 2227 2242 12487353 10.1111/j.0014-3820.2002.tb00147.x

[B31] Duffy JE Species boundaries, specialization, and the radiation of sponge-dwelling alpheid shrimp Biol J Linn Soc 1996 58 307 324

[B32] Knowlton N Jackson JBC New taxonomy and niche partitioning on coral reefs: Jack of all trades or master of some? Trends Ecol Evol 1994 9 7 9 10.1016/0169-5347(94)90224-0 21236753

[B33] Santos SR T.L. Shearer A.R. Hannes Coffroth. MA Fine-scale diversity and specificity in the most prevalent lineage of symbiotic dinoflagellates (Symbiodinium, Dinophyceae) of the Caribbean Mol Ecol 2004 13 459 469 10.1046/j.1365-294X.2003.02058.x 14717900

[B34] Knowlton N Mate JL Guzman HM Rowan R Jara J Direct evidence for reproductive isolation among the three species of the Montastraea annularis complex in Central America (Panama and Honduras) Mar Biol 1997 127 705 711 10.1007/s002270050061

[B35] Lessios HA Possible prezygotic reproductive isolation in sea urchins separated by the Isthmus of Panama Evolution 1984 35 1144 1148 10.2307/2408446 28555797

[B36] Levitan DR Fukami H Jara J Kline D McGovern TA McGhee KM Swanson CA Knowlton N Mechanisms of reproductive isolation among sympatric broadcast-spawning corals of the Montastraea annularis complex Evolution 2004 58 308 323 15068348

[B37] Hellberg ME Moy GW Vacquier VD Positive selection and propeptide repeats promote rapid Interspecific divergence of a gastropod sperm protein Mol Biol Evol 2000 17 3 458 466 10723746 10.1093/oxfordjournals.molbev.a026325

[B38] Palumbi SR Howard DJ, Berlocher SH Species formation and the evolution of gamete recognition loci Endless Forms: Species and Speciation 1998 NY , Oxford Univ. Press 271 278

[B39] Vacquier VD Swanson WJ Lee YH Positive Darwinian selection on two homologous fertilization proteins: what is the selective pressure driving their divergence? J Mol Evol 1997 44 15 22 10.1007/PL00000049 9071007

[B40] Knowlton N Sibling species in the sea Annu Rev Ecol Syst 1993 24 189 216 10.1146/annurev.es.24.110193.001201

[B41] Arnold ML Natural Hybridization and Evolution Oxford Series in Ecology and Evolution 1997 New York , Oxford Univ. Press

[B42] Rieseberg LH Fossen CV Desrochers AM Hybrid speciation accompanied by genomic reorganization in wild comparative mapping between Arabidopsis sunflowers Nature 1995 375 313 316 10.1038/375313a0

[B43] Veron JEN Corals in space and time. Sydney 1995 Sydney , Univ. of New South Wales Press 321

[B44] Kim K Lasker HR Flow-mediated competition among suspension feeding gorgonians J Exp Mar Biol Ecol 1997 215 49 64 10.1016/S0022-0981(97)00015-4

[B45] Skoufas G Comparative biometry of Eunicella singularis (gorgonian) sclerites at East Mediterranean Sea (North Aegean Sea, Greece) Mar Biol 2006 149 1365 1370 10.1007/s00227-006-0314-4

[B46] Bayer FM The Shallow-Water Octocorallia of the West Indian Region. A Manual for marine biologists 1961 The Hague , Martinus Nijhoff 373 pp

[B47] West JM Plasticity in the sclerites of a gorgonian coral: Tests of water motion, light level, and damage cues Biol Bull 1997 192 279 289 10.2307/1542721 28581870

[B48] Kinzie RA The zonation of West Indian gorgonians Bul Mar Sci 1973 23 93 155

[B49] Yoshioka PM Yoshioka BB Effects of water motion, topographic relief and sediment transport on the distribution of shallow-water gorgonian community Mar Ecol Prog Ser 1989 54 257 264 10.3354/meps054257

[B50] Kimura M A simple method for estimating evolutionary rates of base substitutions through comparative studies of nucleotide sequences. J Mol Evol 1980 16 111 120 10.1007/BF01731581 7463489

[B51] Pigliucci M Touchy and bushy: phenotypic plasticity and integration in response to wind stimulation in Arabidopsis thaliana Int J Plant Sci 2002 163 399 408 10.1086/339158

[B52] Okamura BJ Partridge C Suspension Feeding Adaptations to Extreme Flow Environments in a Marine Bryozoan Biol Bull 1999 196 205 215 10.2307/1542566 28296474

[B53] Callahan H Pigliucci M Shade-induced plasticity and its ecological significance in wild populations of Arabidopsis thaliana Ecology 2002 83 1965 1980

[B54] Monro K Poore AGB Light quantity and quality induce shade-avoiding plasticity in a marine macroalga J Evol Biol 2005 18 426 435 10.1111/j.1420-9101.2004.00826.x 15715848

[B55] Graus RR Macintyre. IG Light control of growth forms in colonial reef corals: computer simulation Science 1976 193 895 897 10.1126/science.193.4256.895 17753640

[B56] Grigg RW Orientation and growth form of sea fans Limn Oceanogr 1972 17 185 192

[B57] Lesser MP Weis VM Patterson MP Jokiel PL Effects of morphology and water motion on carbon delivery and productivity in the reef coral, Pocillopora damicornis (Linnaeus): diffusion barriers, inorganic carbon limitation, and biochemical plasticity J Exp Mar Biol Ecol 1994 178 153 179 10.1016/0022-0981(94)90034-5

[B58] Sebens KP Witting J Helmuth B Effects of water flow and branch spacing on particle capture by the reef coral Madracis mirabilis (Duchassaing and Michelotti) J Exp Mar Biol Ecol 1997 211 1 28 10.1016/S0022-0981(96)02636-6

[B59] Meroz E Brickner I Loya Y Peretzman-Shemer A Ilan M The effect of gravity on coral morphology Proc R Soc Lond B 2002 269 717 720 10.1098/rspb.2001.1924 PMC1690943 11934363

[B60] Stambler N Dubinsky Z Corals as light collectors: an integrating sphere approach Coral Reefs 2005 24 1 9 10.1007/s00338-004-0452-4

[B61] de Kroon H H.Huber J.F.Stuefer van Groenendael JM A modular concept of phenotypic plasticity in plants New Phytologist 2005 166 73 82 10.1111/j.1469-8137.2004.01310.x 15760352

[B62] Yoshioka PM Yoshioka BB A comparison of the survivorship and growth of shallow-water gorgonian species of Puerto Rico Mar Ecol Prog Ser 1991 69 253 260 10.3354/meps069253

[B63] McFadden CS France SC Sánchez JA Alderslade P A molecular phylogenetic analysis of the Octocorallia (Cnidaria: Anthozoa) based on mitochondrial protein-coding sequences Mol Phyl Evol 2006 41 513 527 10.1016/j.ympev.2006.06.010 16876445

[B64] Sanchez JA McFadden CS France SC Lasker HR Molecular phylogenetic analyses of shallow-water Caribbean octocorals Mar Biol 2003 142 975 987

[B65] Hellberg ME Dependence of gene flow on geographic distance in two solitary corals with different larval dispersal capabilities Evolution 1996 50 1167 1175 10.2307/2410657 28565289

[B66] Avise JC Molecular Markers, Natural History and Evolution 2004 2nd Sunderland MA , Sinauer 684 p

[B67] Mayr E Systematics and the Origin of Species 1942 New York , Columbia Univ. Press

[B68] Baums IB Miller MW Hellberg ME Regionally isolated populations of an imperiled Caribbean coral, Acropora palmata Molecular Ecology 2005 14 1377 1390 10.1111/j.1365-294X.2005.02489.x 15813778

[B69] Gutiérrez-Rodríguez C Lasker HR Microsatellite variation reveals high levels of genetic variability and population structure in the gorgonian coral Pseudopterogorgia elisabethae across the Bahamas Mol Ecol 2004 13 2211 2221 10.1111/j.1365-294X.2004.02247.x 15245395

[B70] Taylor MS Hellberg ME Marine radiations at small geographic scales: speciation in Neotropical reef gobies (Elacatinus) Evolution 2005 59 374 385 15807422

[B71] Vollmer SV Palumbi SR Restricted gene flow in the Caribbean staghorn coral Acropora cervicornis: Implications for the recovery of endangered reefs J Heredity 2007 98 40 50 10.1093/jhered/esl057 17158464

[B72] Briggs JC Operation of zoogeographic barriers Syst Zool 1973 23 248 256 10.2307/2412136

[B73] Shulman MJ Bermingham E Early life histories, ocean currents, and the population genetics of Caribbean reef fishes Evolution 1995 49 897 910 10.2307/2410412 28564869

[B74] Zardus JD Hadfield MG Multiple origins and incursions of the Atlantic barnacle Chthamalus proteus in the Pacific Mol Ecol 2005 14 12 3719–3733 10.1111/j.1365-294X.2005.02701.x 16202091

[B75] Palumbi SR Marine speciation on a small planet Trends Ecol Evol 1992 7 114 117 10.1016/0169-5347(92)90144-Z 21235975

[B76] Diekmann OE Olsen JL Stam WT Bak RPM Genetic variation within Symbiodinium clade B from the coral genus Madracis in the Caribbean (Netherlands Antilles) Coral Reefs 2003 22 29 33

[B77] Rowan R Knowlton N Intraspecific diversity and ecological zonation in coral-algal symbiosis Proc Natl Acad Sci 1995 92 2850 2853 10.1073/pnas.92.7.2850 7708736 PMC42316

[B78] Rieseberg LH Carter R Zona S Molecular tests of the hypothesized hybrid origin of two diploid Helianthus species (Asteraceae) Evolution 1990 44 1498 1511 10.2307/2409332 28564296

[B79] Pyle RL Randall JE A review of hybridization in marine angelfishes (Perciformes: Pomacanthidae) Environ Biol Fishes 1994 41 127 145

[B80] Schelly R Salzburger W Koblmuller S Duftner N Sturmbauer C Phylogenetic relationships of the lamprologine cichlid genus Lepidiolamprologus (Teleostei: Perciformes) based on mitochondrial and nuclear sequences, suggesting introgressive hybridization. Mol Phyl Evol 2006 38 426 438 10.1016/j.ympev.2005.04.023 15964213

[B81] Gilg MR Hilbish TJ Patterns of larval dispersal and their effect on the maintenance of a blue mussel hybrid zone in Southwest England Evolution 2003 57 1061 1077 12836823 10.1111/j.0014-3820.2003.tb00316.x

[B82] Hatta M Fukami H Wang WQ Omori M Shimoike K Hayashibara T Ina Y Sugiyama T Reproductive and genetic evidence for a reticulate evolutionary history of mass-spawning corals Mol Biol Evol 1999 16 1607 1613 10555292 10.1093/oxfordjournals.molbev.a026073

[B83] van Oppen MJH Willis BL van Vugt HWJA Miller DJ Examination of species boundaries in the Acropora cervicornis group (Scleractinia, Cnidaria) using nuclear DNA sequence analyses Mol Ecol 2000 9 1363 1373 10.1046/j.1365-294x.2000.01010.x 10972775

[B84] Vollmer SV Palumbi SR Hybridization and the evolution of reef coral diversity Science 2002 296 2023 2025 10.1126/science.1069524 12065836

[B85] Kenyon JC Models of reticulate evolution in the coral genus Acropora based on chromosome numbers: parallels with plants Evolution 1997 51 756 767 10.2307/2411152 28568585

[B86] Willis BL van Oppen MJH Miller DJ V. VS Ayre DJ The role of hybridization in the evolution of reef corals Annu Rev Ecol Syst 2006 37 489 517 10.1146/annurev.ecolsys.37.091305.110136

[B87] Burke JM Rieseberg LH The fitness effects of transgenic disease resistance in wild sunflowers Science 2003 300 1250 10.1126/science.1084960 12764188

[B88] Gompert Z Fordyce JA Forister ML Shapiro AM Nice CC Homoploid hybrid speciation in an extreme habitat Science 2006 314 1923 1925 10.1126/science.1135875 17138866

[B89] Sánchez JA Aguilar C Dorado D Manrique N Phenotypic plasticity and morphological integration in a marine modular invertebrate BMC Evolutionary Biology 2007 7 122 10.1186/1471-2148-7-122 17650324 PMC1959521

[B90] Lasker HR Coffroth MA Octocoral distribution at Carrie Bow Cay, Belize Mar Ecol Prog Ser 1983 13 21 28 10.3354/meps013021

[B91] Beiring EA Lasker HR Egg production by colonies of a gorgonian coral Mar Ecol Prog Ser 2000 196 169 177 10.3354/meps196169

[B92] Lasker HR Asexual reproduction, fragmentation, and skeletal morphology of a plexaurid gorgonian Mar Ecol Prog Ser 1984 19 261 268 10.3354/meps019261

[B93] Garcia JR Schmitt C Heberer G Winter A La Parguera Puerto Rico UNESCO. CARICOMP- Caribbean coral reef, sea grass and mangrove sites 1998 Paris , UNESCO 347

[B94] Prada C Phenotypic and genetic variability in the octocoral Plexaura flexuosa. Master's thesis Department of Marine Sciences 2007 M.S. Puerto Rico , University of Puerto Rico, Mayaguez.

[B95] Sokal RR Rohlf FJ Biometry. 3rd ed 1995 New York , W. H. and Freeman

[B96] Quinn GP Keough MJ Experimental design and data analysis for biologists 2002 Cambridge, UK , Cambridge Univ. Press

[B97] Pont-Kingdon GA Okada NA Macfarlane JL Beagley CT Wolstenholme DR Cavalier-Smith T Clark-Walker GD A coral mitochondrial mutS gene Nature 1995 375 109 111 10.1038/375109b0 7753165

[B98] Takabayashi M Carter DA Loh WKW Hoegh-Guldberg O A coral specific primer for PCR amplification of the internal transcribed spacer region in ribosomal DNA Mol Ecol 1998 7 925 931 10.1111/j.1365-294X.1998.00368.x 9691494

[B99] France SC Hoover LL Analysis of variation in mitochondrial DNA sequences (ND3, ND4L, MSH) among Octocorallia (=Alcyonaria) (Cnidaria: Anthozoa) Bull Biol Soc Wash 2001 10 110 118

[B100] Gordon D Baxevanis AD DDB Viewing and editing assembled sequences using consed Current Protocols in Bioinformatics 2004 New York , John Wiley & Co 11.12.11 11.12.43 10.1002/0471250953.bi1102s02 18428695

[B101] Maddison WP Maddison DR MacClade version 3: Analysis of phylogeny and character evolution. (book) + 900K (computer program) 1992 Sunderland MA , Sinauer 398 pp 10.1159/000156416 2606395

[B102] Nei M Molecular evolutionary genetics 1987 New York , Columbia Univ. Press

[B103] Rozas J Sandez-DelBurrio JC Messeguer X Rozas. R DnaSP: DNA polymorphism analyses by the coalescent and other methods Bioinformatics 2003 19 2496 2497 10.1093/bioinformatics/btg359 14668244

[B104] Tajima F Statistical method for testing the neutral mutation hypothesis by DNA polymorphism. Genetics 1989 105 437 460 2513255 10.1093/genetics/123.3.585 PMC1203831

[B105] Fu YX Statistical tests of neutrality of mutations against population growth, hitchhiking and background selection Genetics 1997 147 2 915 925 9335623 10.1093/genetics/147.2.915 PMC1208208

[B106] Schneider S Roessli D Excoffier L ARLEQUIN. A software for population genetic data analysis, Version 2.00 2000 Geneva, Switzerland , Genetics and Biometry Laboratory, Department of Anthropology, Univ. of Geneva

[B107] Templeton AR Crandall KA Sing CF A cladistic analysis of phenotypic associations with haplotypes inferred from restriction endonuclease mapping and DNA sequence data. III. Cladogram estimation. Genetics 1992 132 619 633 1385266 10.1093/genetics/132.2.619 PMC1205162

[B108] Clement M Posada D Crandall K TCS: a computer program to estimate gene genealogies Mol Ecol 2000 9(10) 1657 1660 10.1046/j.1365-294x.2000.01020.x 11050560

[B109] Excoffier L Smouse PE Quattro JM Analysis of molecular variance inferred from metric distances among DNA haplotypes: application to human mitochondrial restriction data Genetics 1992 131 479 491 1644282 10.1093/genetics/131.2.479 PMC1205020

[B110] Swofford DL PAUP*: Phylogenetic analysis using parsimony*, version 4.0b10 2002 Sunderland, MA , Sinauer

[B111] Posada D Crandall KA Modeltest: testing the model of DNA substitution Bioinformatics 1998 14 817 818 10.1093/bioinformatics/14.9.817 9918953

[B112] Hasegawa M Kishino H Yano T Dating of the human-ape splitting by a molecular clock of mitochondrial DNA J Mol Evol 1985 22 160 174 10.1007/BF02101694 3934395

[B113] McGehee A Comparisons of water motion in coral reef by measuring corrosion rates of dissimilar metals. Carib J Sci 1998 34 286 297

